# Security Enhanced EMV-Based Mobile Payment Protocol

**DOI:** 10.1155/2014/864571

**Published:** 2014-09-15

**Authors:** Ming-Hour Yang

**Affiliations:** Department of Information & Computer Engineering, Chung Yuan Christian University, 200 Chung Pei Road, Chung Li, Taoyuan County 32023, Taiwan

## Abstract

Near field communication has enabled customers to put their credit cards into a smartphone and use the phone for credit card transaction. But EMV contactless payment allows unauthorized readers to access credit cards. Besides, in offline transaction, a merchant's reader cannot verify whether a card has been revoked. Therefore, we propose an EMV-compatible payment protocol to mitigate the transaction risk. And our modifications to the EMV standard are transparent to merchants and users. We also encrypt the communications between a card and a reader to prevent eavesdropping on sensitive data. The protocol is able to resist impersonation attacks and to avoid the security threats in EMV. In offline transactions, our scheme requires a user to apply for a temporary offline certificate in advance. With the certificate, banks no longer need to lower customer's credits for risk control, and users can have online-equivalent credits in offline transactions.

## 1. Introduction

Since credit cards can be embedded with radio frequency identification (RFID) tags, such as MasterCard's PayPass [1] and Visa's payWave [2], contactless payment has brought much convenience in shopping. However, behind all of the convenience of these new e-commerce products lie certain security threats. For instance, some security problems have been found in MIFARE Classic RFID tags [3, 4].

For securing the execution environment on a mobile device, Ekberg and Bugiel [5] shrink the mobile trusted module (MTM) to fit the secure element (SE) of a near field communication (NFC) embedded cellular phone. For making heterogeneous MTM, Winter [6] proposes an ARM TrustZone's virtualization technique to operate MTM on Linux. Nauman et al. [7] use the secure boot scheme on MTM to authenticate software's origin and therefore can detect malware on android systems. This technique can be applied to protect users' sensitive data from malware on an NFC phone and prevents data leakage, for example, users' credit card information in Google Wallet [8] and in Microsoft's multiple virtual credit cards, which are remotely created by the trusted platform module virtual smart card (TPMVSC) [9]. These improvements provide higher mobile system security and more convenience for current mobile payment services.

Apart from protecting sensitive data and transaction agents on the mobile devices, some mobile payment protocols are proposed to secure financial transactions [10–15]. To comply with micropayment infrastructure, Chen et al. [11] and Ali and Awal [10] propose their NFC micropayment schemes based on GSM networks. Later Chen et al. come up with a new scheme [12] combining NFC and 3G into mobile payment services. These methods use the temporary mobile subscriber identity (TMSI) and the telecom-user shared keys for authentication. Because these methods have to be tied up with ISPs and lack flexibility, Chen et al. [13] and Mainetti et al. [15] use certificates to authenticate the subscribers. But, none of them conforms to current credit card payment standards, such as PayPass Magstripe [16], Visa MSD [2], and Europay MasterCard Visa (EMV) [17].

Google Wallet [8] runs a credit card transaction protocol PayPass Magstripe [16] on NFC-embedded phones to provide payment services. But PayPass Magstripe does not have a machine-to-machine client end nonrepudiation mechanism. Pasquet et al. [18] implement a proved nonrepudiation mobile payment standard PayPass M/chip [1, 19] on NFC phones to automatically prevent customers' denial of their transactions. Besides, WatchData implements Zuo's Dual Interface Sim into a product called SIMpass [20]. It allows non-NFC smartphones to run PBOC 2.0, aka the Chinese EMV standards.

Because a point of sale (POS) may not connect its back-end server, offline transaction services are required, for example, transactions on an airplane. During offline sessions, a merchant cannot check if a credit card has been revoked. A malicious customer can perform transactions with a legitimate but revoked credit card [21]. Hence, prepaid cards like e-cash [22–24] and e-voucher [25, 26] are used to lower the chance of credit card fraud in offline transactions. Damme et al. [25] propose a method to prepay a voucher for future transactions. The voucher is encrypted by its issuer and is stored on the SE of a user's NFC phone. But this method is impractical because its subscribers have to predict the amount of money that their future shopping will take.

Instead of amount prediction, EMV sets the following three rules for its risk management [17] requiring an offline transaction to go online or to be aborted: (1) when the cumulative amount of offline transactions exceeds the threshold; (2) when the consecutive number of offline transactions is over the maximum times; (3) when the current transaction amount exceeds the random descending threshold. However, a malicious user may forge the counter of consecutive offline transactions to evade the second rule [27].

To prevent forgery of threshold values, Blaze et al. propose a certificate-based risk management scheme [28], whose certificate contains the threshold of cumulative amount and the threshold of consecutive number of offline transactions. Rivest and Shamir propose PayWord [29] to limit the amount of a customer's cumulative paywords, which must be smaller than the credit limit written in the bank-issued certificate. Fan and Liao [30] propose the use of master-slave paywords to decrease a user's risk in a transaction. Because a user may not receive his service/product that he has paid for, they propose that the user sends his master payword to the merchant after receiving the service. Furthermore, Fan et al. [31] use one-time certificates in their postpaid method to improve the master-slave payword scheme [30] by providing user anonymity. However, in a mobile payment environment, to generate paywords for offline transactions is impractical because users' mobile devices have only limited computing ability. Also, it is difficult for users to generate a payword in advance because they cannot predict the amount of their future transactions. So, the Payword schemes may not be suitable for mobile payment [32]. What is worse is that these Payword schemes can cause double spending if a user takes a payword to different shops before the previous merchant can cash the payword [28, 32].

We propose an EMV-compatible mobile payment protocol, in which our modifications to the original EMV standards are transparent to merchants and users. Our payment protocol is based on the current credit card transaction infrastructure and it uses EMV commands' reserved bits and optional fields to transfer additional information. Therefore, a merchant can simply patch his reader's payment program to run our protocol. Besides, our protocol verifies each offline transaction using a bank-issued temporary risk management certificate in the users' NFC phones. The users need to apply for the certificate from their issuing bank for the upcoming offline transactions. To avoid inconvenience in the certificate application, the certificate can be transferred to the customer on his way to the offline shopping area. For instance, when a customer purchases a flight ticket at an airport's check-in counter, the airline can write the certificate into his NFC phone simultaneously for his later in-flight offline shopping. Therefore, the bank can write its risk management rules into the certificate, such as the list of authorized merchants in the offline shopping area, the customer's credit limit, and the expiry date of the card. We prove that our offline transaction risk is equivalent to the risk in an online transaction. Besides, our mobile payment protocol prevents the potential threats found in EMV online transactions as follows.

1. Our mobile payment protocol averts double spending.
2. Our protocol is secure against the Man-in-the-Middle attacks that evade PIN verification as discussed in Murdoch et al.'s scheme [33].
3. We propose a mutual authentication protocol to prevent an unauthorized reader from retrieving the credit card's information [33, 34].
4. Sensitive information is encrypted to prevent information leakage in NFC's phone-reader communications [35, 36]. Such information leakage can cause credit card fraud in online transactions [37] and in offline transactions [19, 33].
5. Our protocol avoids the card cloning attack [27]. When an attacker eavesdrops on the random number and amount in a user's transaction messages, he cannot use the sniffed information to perform a preplay attack in his latter transactions.

The details of our scheme will be provided in Section 2. We will analyze its security and performance in Sections 3 and 4. A conclusion and the future scope will be drawn in Section 5.

## 2. Security Enhanced EMV-Based Mobile Payment Protocol

**Figure 1:**
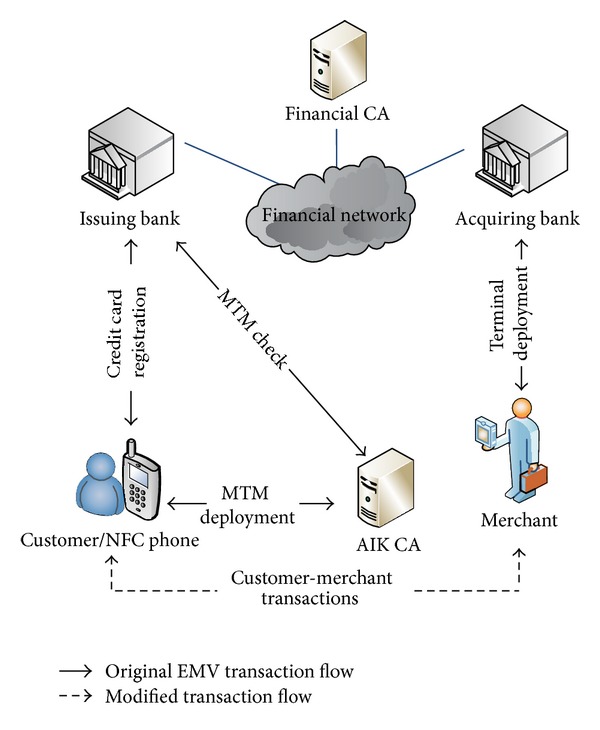
Our credit card transaction infrastructure.

We propose an EMV-compatible mobile payment protocol for users to perform online and offline transactions with the credit cards stored in their mobile devices. The protocol is based on current credit card transaction infrastructure, as shown in Figure 1. The original transaction flow remains unchanged, except the user-merchant communications; see the dotted lines in Figure 1. In our user-merchant communications, some messages are inserted into the original EMV transaction flow to read information from the mobile phone for mutual authentication.

Besides, EMV commands' optional fields are also used to transmit a bank-issued certificate so as to control the risk in offline transactions. Our environment assumptions for the financial network are the same as the original EMV's:

- the bank that issues credit cards communicates with the bank that deploys POS terminals through a secured financial network;
- in offline transactions, a POS terminal cannot connect its acquiring bank to check if a credit card has been revoked.

**Figure 2:**
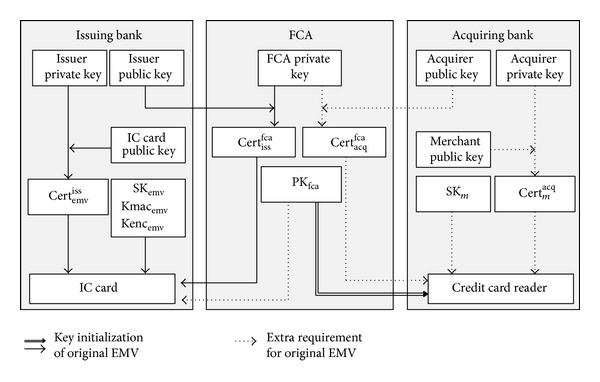
Key initialization of cards and card readers in our scheme.

The POS's card reader, as the double line depicts in Figure 2, is initialized with the public key PK_fca_ of the financial certificate authority (FCA) and it communicates with its acquiring bank through a secured channel. The POS cannot be compromised by adversaries.

We have to note that the interbank communications in financial networks are protected with their existing secure mechanisms and this part is beyond the scope of this paper. Because the focus of this protocol is on the contactless credit card payment, in the rest of this paper we will use a card reader to represent a POS terminal.

As the dotted lines show in Figure 2, for authenticating a merchant's card reader in offline transactions, each reader is initialized with SK_*m*_, Cert_*m*_^acq^, and Cert_acq_^fca^. SK_*m*_ is the merchant's private key. Cert_*m*_^acq^ is the certificate that an acquiring bank issues to its merchants. Cert_acq_^fca^ is the certificate that FCA issues to the acquiring bank. Since the payment agent does not run on a traditional smart card, we need to protect the payment protocol from the interference of other programs running on the smartphone. Therefore, the assumptions for our client end are defined as follows.

- A user's phone is NFC-enabled and has a secure element, which is powered by MTM [38], so as to provide a secure memory and execution environment for the client end payment program.
- The private key SK_*p*_ and the certificate Cert_*p*_^aik^ can be securely distributed to the phone's secure element [5, 6, 8, 39, 40]. These two items can be written into the phone by its manufacturer [8] or distributed by the bank and the service provider through their current secure channels [39, 40].

The notations used in our scheme are listed in the Notations.

When a user applies for a credit card from an issuing bank, he needs to use the private key SK_*p*_ and the certificate Cert_*p*_^aik^, which are distributed into his phone during initialization, to pass the authentication. Further, he is authorized to download the payment agent and set the agent's access code. Finally, the issuing bank writes the following EMV-required data into the user's phone to create a virtual credit card [41, 42].

- Data_emv_ = {PAN, EX_DATE, CDOL1, CDOL2, SSAD,…} is a set of required data for credit card transactions [17].
- With the function of MTM's monotonic counter [43], the credit card's application transaction counter (ATC) is set to 0.
- Two shared keys Kmac_emv_ and Kenc_emv_, one private key SK_emv_, and two certificates Cert_emv_^iss^ and Cert_iss_^fca^ are generated by the issuing bank.

Here Data_emv_ only lists the minimum requirements for EMV credit card transaction [17]: the card's primary account number (PAN); the card's expiry date EX_DATE; card risk management data object lists CDOL1 and CDOL2; and the issuing bank's signed static application data (SSAD), which is a signed hash value of PAN, EX_DATE, CDOL1, and CDOL2. When an issuing bank adds some customized rules into Data_emv_, a card reader follows our protocol to retrieve the minimum information from a credit card, and it follows the original EMV protocol to retrieve the bank-required information, such as the country code. Since the banks' customized rules will not affect our protocol, our discussion will only focus on the minimum sets of Data_emv_.

Besides, our payment protocol requires FCA's public key PK_fca_ to be written into the phone to authenticate the merchants:

 FCA's public key PK_fca_.

So, a phone will be issued a virtual credit card card_emv_ when the issuing bank approves the application:

(1) cardemv={Dataemv,ATC,Kmacemv,Kencemv,  SKemv,Certemviss,Certissfca,PKfca}.

The virtual credit card will be stored on the phone as one of the cards in the card set cards:

(2) $$\begin{array}{c} \text{cards}=\text{cards}\cup {\text{card}}_{\text{emv}}. \end{array}$$

If a user takes his phone's SK_*p*_ and Cert_*p*_^aik^ to apply for other *n* credit cards, there will be card_emv1_, card_emv2_,…, card_emv*n*_ stored on the phone.

cards = {(cards ∪ card_emv*i*_)∣*i* = 1, 2,…, *n*} will be stored eventually as follows:

(3) cards={{Dataemv,ATC,Kmacemv,Kencemv,SKemv,  Certemviss,Certissfca,PKfca},{Dataemv1,ATC1,Kmacemv1,Kencemv1,SKemv1,Certemv1iss1,Certiss1fca,PKfca},…,{Dataemvn,ATCn,Kmacemvn,Kencemvn,SKemvn,Certemvnissn,Certissnfca,PKfca}}.

Since all the virtual credit cards in the set cards are stored on the phone, their transaction behaviors are identical. Without loss of generality we use the card card_emv_ to represent a virtual card in the cards in our following discussion.

**Figure 3:**
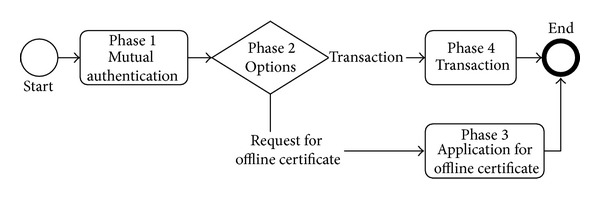
Flow chart of our protocol.

As Figure 3 shows, our EMV-based online/offline transaction protocol consists of four phases. The first is a user-merchant mutual authentication protocol. The second phase decides whether the user needs an offline certificate or he needs a transaction service. If the user visits a card reader that is designed for issuing the offline certificate and his phone does not have a valid certificate, our protocol enters the third phase and writes a temporary certificate into his phone automatically. For instance, when a user is in his check-in service at an airline counter, the reader on the counter can write a bank-issued offline certificate into his NFC phone simultaneously. With the certificate, the user is enabled to do offline shopping on board.

If the user's certificate is still valid or he only needs online transaction, the protocol goes to the last phase to complete the payment. If the reader is online, it will run our online transaction protocol; otherwise the reader runs our offline transaction protocol to check the offline certificate first to lower the offline transaction risk.

**Figure 4:**
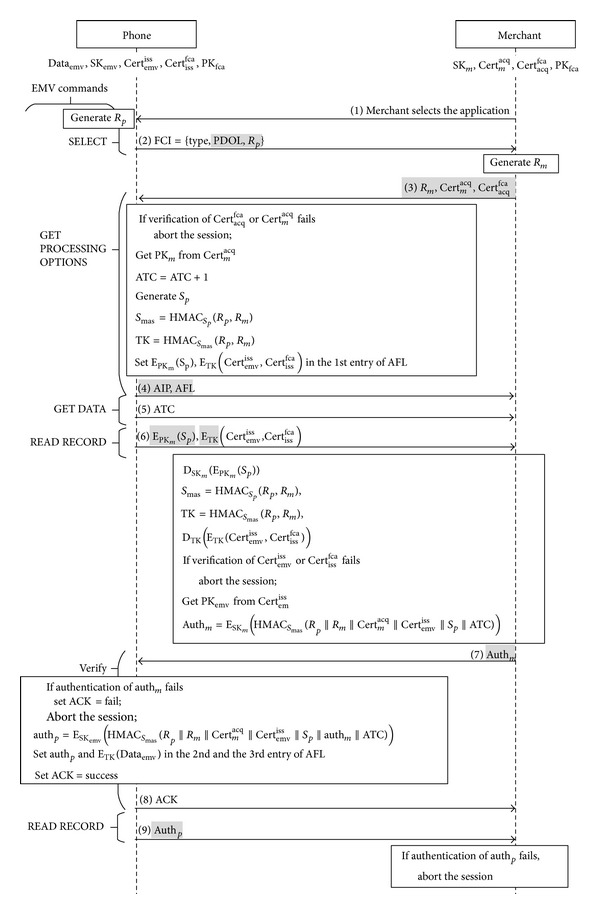
Mutual authentication (modifications are highlighted).

Before we go into the details of our payment protocol, we have to define each entity used in the diagrams of our protocol flows. Take Figure 4 as an example. The boxes on the top indicate the role of each correspondence and their initialized data. On the left side of the diagram are the EMV commands. Each command is sent by a merchant and responded by a virtual credit card on the phone. In order to demonstrate our protocol concisely, we only show returned messages for the two commands: READ RECORD and GET DATA. The merchant sends these two commands to a credit card just for data retrieval, so we do not show this part in our figures. Above each arrow line we indicate the messages to be sent. And each box below a line shows the actions to be taken by the receiver after the message is received. To highlight the difference between our protocol and the original EMV transaction processing, we use grey-patterned messages to refer to the modified messages.

**Phase 1 (mutual authentication).**

Once the access code is verified, a user can unlock the protected credit card keys and data [44] and start the agent to run the client end payment protocol, as Figure 4 shows. The agent responds to the merchant's EMV command SELECT with file control information (FCI). Consider FCI = {type, PDOL, *R*_*p*_}, where the required message type denotes the card type and FCI's format. The optional tag PDOL is used to indicate what data the reader needs to send back to the phone in the next command GET PROCESSING OPTIONS. Besides, we create a new tag for the command SELECT [17], so that the phone is able to send the random number *R*_*p*_ to the reader.

*Message 3-4*. After receiving FCI from the phone, the reader has to follow PDOL's indication to return the following information to the phone: a random number *R*_*m*_, the merchant's certificate Cert_*m*_^acq^, and the acquiring bank's certificate Cert_acq_^fca^. Since these three parameters are not defined in EMV, we need to create three new tags for the command GET PROCESSING OPTIONS [17] to return the PDOL-required information.

When the phone receives the PDOL-required message, it uses the public key PK_fca_ to verify Cert_acq_^fca^ and takes the acquiring bank's public key PK_acq_ to verify Cert_*m*_^acq^. If the verification fails, the phone will abort the transaction. If the two certificates are verified, the phone will take the following 6 steps to send the application interchange profile (AIP) and the application file locator (AFL) back to the reader:

- it increases the transaction counter ATC by 1;
- it retrieves the merchant's public key PK_*m*_ from Cert_*m*_^acq^;
- it uses a random number *S*_*p*_ as the key to hash random numbers *R*_*p*_ and *R*_*m*_ so as to create the session key TK;
- it writes the encrypted secret *E*_PK_*m*__(*S*_*p*_) and the encrypted certificates *E*_TK_(Cert_emv_^iss^, Cert_iss_^fca^) into the first memory address in AFL;
- it sets a flag in AIP to indicate that the credit card supports our mutual authentication protocol;
- it sends AFL and AIP to the reader.

*Message 5*. When the reader receives AIP indicating that the credit card supports our mutual authentication scheme, it sends the command GET DATA to retrieve the credit card's transaction counter ATC.

*Message 6*. After receiving the card's ATC, the reader retrieves the first memory address from AFL and sends the command READ RECORD with the address to retrieve the data set *E*_PK_*m*__(*S*_*p*_) and *E*_TK_(Cert_emv_^iss^, Cert_iss_^fca^) from the phone. The data set has been written into the first memory address in AFL in Message 3.

*Message 7-8.* When the reader receives the encrypted certificates, it retrieves the secret value *S*_*p*_ by using its private key SK_*m*_ to decrypt *E*_PK_*m*__(*S*_*p*_). It takes the secret value as a key to hash random numbers *R*_*p*_ and *R*_*m*_ to create the key *S*_mas_ = HMAC_*S*_*p*__(*R*_*p*_, *R*_*m*_). Then it uses the key *S*_mas_ to generate session key TK. Next it takes the session key TK to decrypt *E*_TK_(Cert_emv_^iss^, Cert_iss_^fca^). It verifies the source of certificates Cert_iss_^fca^ and Cert_emv_^iss^ with the public key PK_fca_. If they are verified, the reader will do the following actions:

(i) it retrieves the public key PK_emv_ from Cert_emv_^iss^;
(ii) it uses the secret value *S*_mas_ to hash all the previously received information as a message authentication code (MAC); finally it takes his private key SK_*m*_ to encrypt the MAC as
  (4) authm =ESKm(HMACSmas(Rp||Rm||Certmacq||Certemviss||Sp||ATC));
(iii) it uses the reserved-for-future-use (RFU) tags of command VERIFY to return auth_*m*_ for the phone to authenticate the reader.

After decrypting the received message with the merchant's public key PK_*m*_, the phone uses the secret *S*_mas_ and all the previously received messages to check if the calculated MAC equals the received MAC. This allows the phone to authenticate the reader because only the reader has the private key SK_*m*_ and the reader has to be the one involved from the beginning of the protocol. If the reader is authenticated, the phone proceeds as follows:

(i) it uses the secret *S*_mas_ to hash all the previously received messages so as to generate a MAC. Then the phone takes its private key SK_emv_ to encrypt the MAC as
  (5) authp=ESKemv (HMACSmas(Rp||Rm||Certmacq||Certemviss||Sp||authm||ATC));
(ii) it uses TK to encrypt Data_emv_;
(iii) it writes auth_*p*_ and *E*_TK_(Data_emv_) into the second and third memory addresses in AFL;
(iv) it returns an acknowledgement to confirm successful authentication.

Otherwise, the phone returns an acknowledgement for failed authentication and aborts the transaction.

*Message 9.* After the reader is authenticated by the phone and the acknowledgement is received, the reader is able to send the command READ RECORD with the second memory address of AFL as a pointer to retrieve auth_*p*_ from the card. The reader decrypts auth_*p*_ with the public key PK_emv_ and uses all the received messages to verify if the calculated auth_*p*_ equals the received auth_*p*_. If these two match, the credit card is authenticated and the reader confirms that this is the card involved from the beginning of the protocol. Otherwise, the reader aborts the transaction.

After mutual authentication, the phone and the reader have verified each other's identity. In the next phase, the protocol will decide the transaction mode according to the configurations of the merchant's reader.

**Phase 2 (choosing transaction mode).**

After the credit card is authenticated by the reader, the reader sends the command READ RECORD with the third memory address of AFL as a pointer to retrieve *E*_TK_(Data_emv_) from the card.

**Figure 5:**
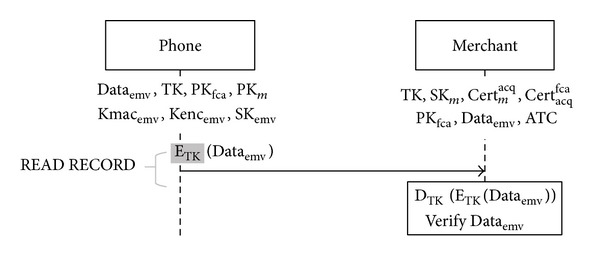
Merchant retrieves data from credit card (modifications are highlighted).

As Figure 5 shows, after receiving *E*_TK_(Data_emv_), the reader decrypts it with the session key TK. The decision-making protocol follows the original EMV standard using static data authentication (SDA) [17] and the bank's SSAD to check the integrity of Data_emv_. But in the case of offline transactions, our protocol needs to check the validity of the credit card's offline certificate Cert_emv_*off*⁡_^iss^ and the transaction time, location, and amount. Therefore, we have to write the check results into EMV's two reserved-for-future-use (RFU) tags in terminal verification results' (TVR) fourth byte [17].

**Figure 6:**
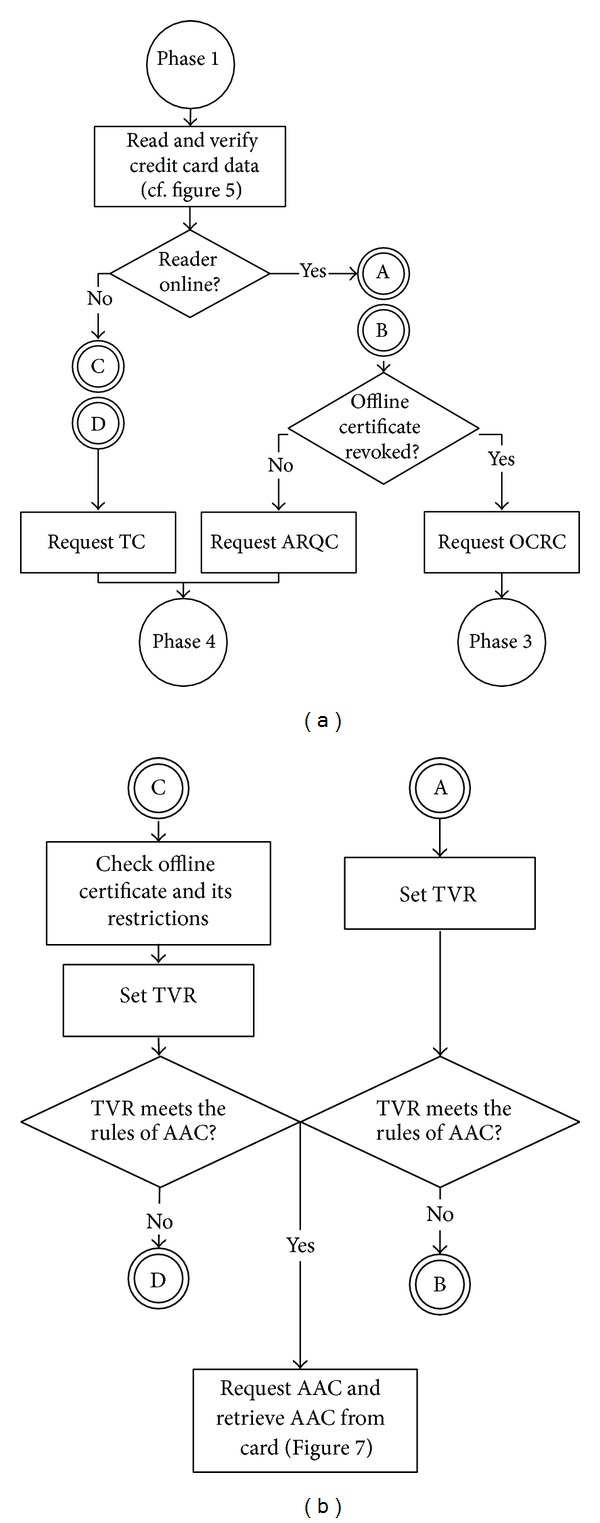
(a) Flow chart of online transaction/offline transaction/request for offline certificate. (b) Flow chart of refusal of transaction.

According to the transaction flow in Figure 6, if a merchant's reader is online, the protocol proceeds to the circle “A” (see Figure 6(b)) to set TVR, as mentioned above. Then it verifies whether TVR meets the rules of EMV's application authentication cryptogram (AAC). If yes, the transaction is aborted. If no, the protocol proceeds to the circle “B” (see Figure 6(b)) to check whether a user's offline certificate has been revoked. If it is not revoked, the protocol proceeds to Phase 4 for online transactions. If the certificate has been revoked, the protocol proceeds to Phase 3 applying for an offline certificate. However, if the reader is not online, the protocol proceeds to the circle “C” (see Figure 6(b)) to check the offline certificate's validity and its restrictions. If the certificate is verified and does not satisfy AAC's rules, the protocol proceeds to the circle “D” (see Figure 6(a)) and the protocol proceeds to Phase 4 for offline transactions.

**Figure 7:**
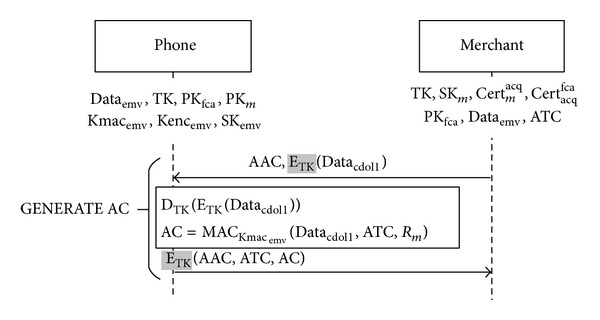
Rejection of transaction in EMV standard (modifications are highlighted).

When a reader decides to abort the transaction, it writes into Data_cdol1_ the data that the issuing bank requires a reader to retrieve from a card. The reader encrypts Data_cdol1_ with TK. Then it follows the original EMV standard using the command GENERATE AC to send EMV's AAC and the encrypted Data_cdol1_ to the credit card; see Figure 7. The card decrypts the message with TK and retrieves Data_cdol1_. Also it uses the user-issuer shared key Kmac_emv_ to hash the received Data_cdol1_, ATC, and *R*_*m*_. The hashed value is used as an authentication code AC⁡ = MAC_Kmac_emv__(Data_cdol1_, ATC, *R*_*m*_). Last, the card encrypts AAC, ATC, and AC with TK and returns the encrypted message to the reader.

As the circle “B” in Figure 6(a) shows, a credit card's Data_emv_ is verified (cf. Figure 5); the card satisfies the issuing bank's rules for risk management, but the credit card does not have a valid offline certificate. In such a case, the reader needs to initiate the process to deploy an offline certificate into the card. However, the original EMV does not have any parameters to activate certificate deployment. Hence, our proposed scheme uses an EMV's RFU as a flag, called offline certificate request cryptogram (OCRC). The flag enables the reader to deploy an offline certificate into a user's phone through the issuing bank; see Phase 3.

As the circle “B” in Figure 6(a) shows, if a reader is online and EMV's TVR indicates that the transaction is approved, the reader requests a user's phone to generate an authorization request cryptogram (ARQC). As the circle “D” in Figure 6(a) shows, if a reader cannot connect its acquiring bank, it has to check whether TVR's offline certificate has expired and whether the transaction time, place, and amount satisfy the certificate's requirements. If they are all verified, the reader requires the phone to generate a transaction certificate (TC), hence the beginning of an offline transaction.

In following paragraphs, we will explain in detail how to apply for an offline certificate.

**Phase 3 (deployment of offline certificate).**

**Figure 8:**
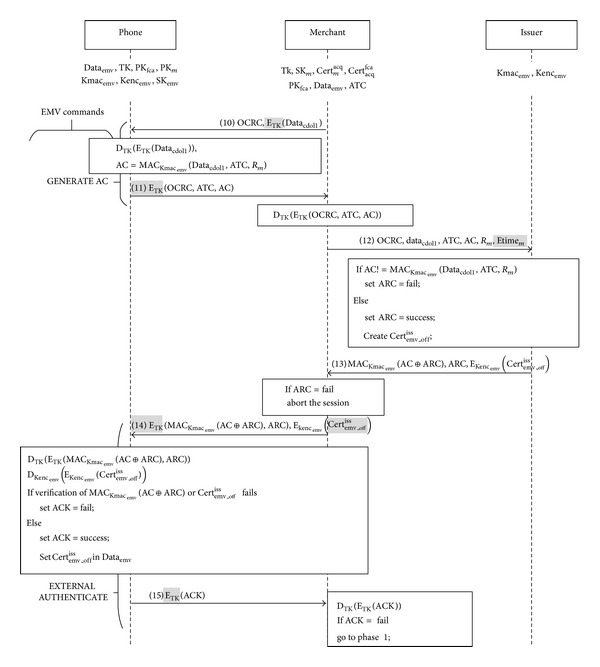
Deployment of offline certificate (modifications are highlighted).

As Figure 8 shows, when a user's phone receives the command GENERATE AC with the parameter OCRC from a merchant's reader, the phone decrypts *E*_TK_(Data_cdol1_).

Then following the EMV standard, the phone uses Kmac_emv_ as the key to hash Data_cdol1_, the credit card's current ATC, and the random number *R*_*m*_, so as to generate AC⁡ = MAC_Kmac_emv__(Data_cdol1_, ATC, *R*_*m*_). Next, the phone uses the session key TK to encrypt OCRC, ATC, and AC. It returns the encrypted message to the reader to begin the deployment of an offline certificate.

*Message 12.* The reader uses TK to decrypt *E*_TK_(OCRC, ATC, AC⁡) and retrieves OCRC, ATC, and AC⁡. Apart from the EMV-required data, that is, OCRC, ATC, AC⁡, Data_cdol1_, and *R*_*m*_, our protocol also requires a reader to send Etime_*m*_ for the issuing bank to set the expiry time of the offline certificate, which is an estimated time to indicate when a user is going to leave the offline shopping area, for example, the arrival time of a flight or the closing time of an exhibition.

*Message 13.* When the issuing bank receives the parameter OCRC for an offline certificate, it takes the shared key Kmac_emv_ to hash the received Data_cdol1_, ATC, and *R*_*m*_, so as to generate MAC_Kmac_emv__(Data_cdol1_, ATC, *R*_*m*_). It checks the data integrity by verifying whether the result equals the received AC⁡. If these two do not match, the bank sets the authorization response code (ARC) as “fail.” Otherwise, it sets ARC = success. Then the bank sets the certificate's expiry time as Etime_*m*_ and restrains the transaction's maximum amount within the user's credit limit. It also restrains user's transaction areas [28] when issuing an offline certificate. For example, an airline's in-flight shopping service can be written into an offline certificate as an authorized merchant. Because the certificate Cert_emv_^iss^ has already included the issuing bank's public key, an offline certificate does not need to include the key. Last, the issuing bank generates an offline certificate, which includes the following:

- identity of the issuer;
- credit card's primary account number (PAN);
- expiry time of the offline certificate;
- maximum sum of transaction amounts before online connection;
- list of authorized merchants in the shopping area.

According to the EMV standard, the issuing bank uses the shared key Kmac_emv_ to generate a message authentication code MAC_Kmac_emv__(AC⁡⊕ARC). It also takes the shared key Kenc_emv_ to encrypt the offline certificate Cert_emv_*off*⁡_^iss^. Next it sends the message authentication code, ARC, and the encrypted certificate to the reader.

*Messages 14-15.* When the reader receives ARC that indicates a fail, it aborts the session. Otherwise, it uses the command EXTERAL AUTHENTICATE's RFU tags to send to a user's credit card the offline certificate *E*_Kenc_emv__(Cert_emv_*off*⁡_^iss^) and the EMV-required information MAC_Kmac_emv__(AC⁡⊕ARC) and ARC. Then the card decrypts the message *E*_TK_(MAC_Kmac_emv__(AC⁡⊕ARC), ARC) with TK. It uses the received ARC, the calculated AC⁡, and the shared key Kmac_emv_ to compute MAC_Kmac_emv__(AC⁡⊕ARC), so as to check the message integrity. Further, it uses Kmac_emv_ to decrypt the offline certificate and to verify the sender of the certificate. If the message integrity and certificate sender are verified, the card writes the certificate into the address that is reserved for an offline certificate in Data_emv_. Hence, Data_emv_ = Data_emv_ ∪ Cert_emv_*off*⁡_^iss^. If the verification fails, the card sets ACK = fail. Last of all, the card uses TK to encrypt ACK to the reader.

The whole process of our offline certificate deployment complies with the original EMV standard. For user transparency, the offline certificate can be written into a user's phone before he enters a designated offline shopping area, for example, at an airline's check-in counter.

**Phase 4 (transaction).**

**Figure 9:**
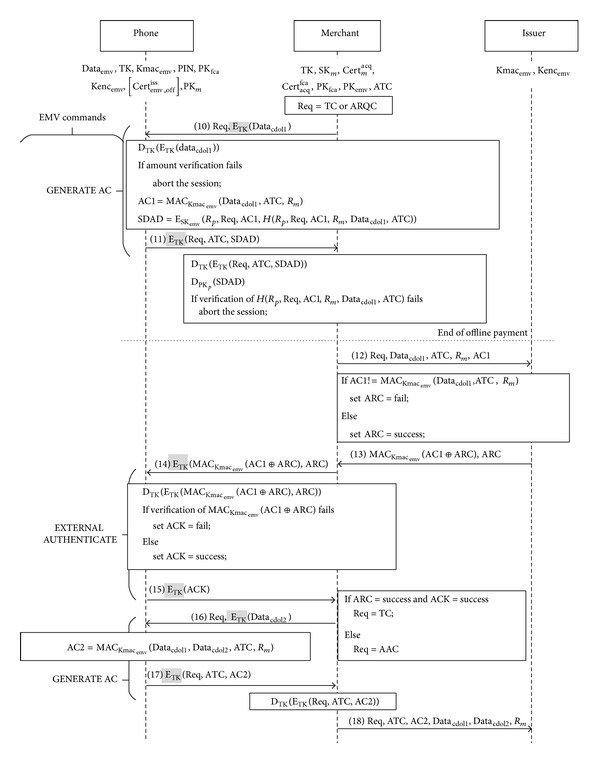
Transaction stage (modifications are highlighted).

In Figure 9, a user's phone receives the command GENERATE AC with the parameters Req (a request for online or offline transaction) and the TK-encrypted Data_cdol1_ from a merchant's reader. If Req = ARQC, the transaction is going online; if Req = TC, the transaction is going offline. As Figure 9 depicts, our proposed transaction protocol complies with the EMV credit card transaction, except that all the user-merchant communications are encrypted with the session key TK.

*Messages 10-11.* After receiving the command, the card decrypts *E*_TK_(Data_cdol1_) with TK. Data_cdol1_'s transaction amount will be displayed on the phone screen. If the user finds the amount incorrect, the transaction is aborted. Otherwise, the card proceeds to the following steps to generate the EMV-required messages.

- The card uses its shared key Kmac_emv_ to hash Data_cdol1_, ATC, and *R*_*m*_ to generate AC1 = MAC_Kmac_emv__(Data_cdol1_, ATC, *R*_*m*_).
- The phone hashes *R*_*p*_, Req, AC1, *R*_*m*_, Data_cdol1_, and ATC, where Req indicates the type of AC1.
- The phone follows the EMV standard and uses its private key to encrypt *R*_*p*_, Req, AC1, and the hash result *H*(*R*_*p*_, Req, AC1, *R*_*m*_, Data_cdol1_, ATC), so as to generate the signed dynamic application data (SDAD).

Last of all, it uses TK to encrypt Req, ATC, and SDAD and sends the encryption to the reader.

*Messages 12-13.* After the reader decrypts the received *E*_TK_(Req, ATC, SDAD) with TK, it uses the card's public key PK_emv_ to decrypt SDAD and verifies the integrity of the message. If the verification fails, it aborts the transaction. If the integrity is verified and Req = TC, the reader follows the original EMV standard to complete the offline transaction with the issuing bank. If Req = ARQC, the reader sends the EMV-required messages Req, Data_cdol1_, ATC, *R*_*m*_, and AC1 to the issuing bank for online transaction. The bank also uses the shared key Kmac_emv_, the received Data_cdol1_, ATC, and *R*_*m*_ to compute MAC_Kmac_emv__(Data_cdol1_, ATC, *R*_*m*_) and verifies the integrity of AC1. If the message integrity is not verified or a credit card transaction cannot pass the evaluation, the bank declines this transaction and sets ARC = fail. Otherwise, it sets ARC = success. Next the bank sets its decision into ARC and sends it with the message authentication code MAC_Kmac_emv__(AC⁡⊕ARC) to the reader.

*Messages 14-15.* The reader uses the command EXTRNAL AUTHENTICATE to forward the TK-encrypted bank decision ARC to the credit card. The card decrypts the message with TK and checks integrity of the received messages. If there is integrity error, ACK is set as “fail.” Otherwise, ACK = success. Last ACK is encrypted with TK and is returned to the reader.

*Messages 16-17.* If the received ACK is success and the received ARC is also success (cf. Message 13), the reader sets Req = TC to indicate that the transaction is complete. Otherwise, it sets Req = AAC to indicate that the transaction is declined. Then the reader uses the second GENERATE AC to send Req and the encryption *E*_TK_(Data_cdol2_) to the credit card. After receiving the command, the card follows the EMV standard using its shared key Kmac_emv_ to hash Data_cdol1_, Data_cdol2_, ATC, and *R*_*m*_, so as to generate AC2 = MAC_Kmac_emv__(Data_cdol1_, Data_cdol2_, ATC, *R*_*m*_). Last of all, the card uses TK to encrypt Req, ATC, and AC2 to the reader.

*Message 18.* After decrypting *E*_TK_(Req, ATC, AC2), the reader follows the EMV standard sending the required messages to the issuing bank to complete the online transaction.

For user transparency, our reader and the issuing bank completely follow the original EMV transaction flow to perform an online/offline transaction or to carry out offline certificate acquisition. In the next section, we will show that our protocol is able to resist most of the security threats in credit card transactions and that we can keep the risk of offline transaction equivalent to that of online transaction.

## 3. Security Analysis

An NFC-enabled phone's secure element (SE) provides secure memory and a safe execution environment for the client end payment program. Besides, the interbank and the reader-bank communications are all protected by their existing secure mechanisms. So, our security analysis will be focused on the potential threats between a reader and a credit card. And we will prove that our offline transaction risk is equivalent to that in an online transaction.

1. Offline mutual authentication: according to our environment assumptions, both a credit card and a reader have their FCA's public key PK_fca_. After receiving an offline certificate from the reader, the card can use the public keys PK_fca_ and PK_acq_ to verify the sources of Cert_acq_^fca^ and Cert_*m*_^acq^, respectively, and then retrieves the reader's public key PK_*m*_. Likewise the reader verifies the certificates and then retrieves the card's public key PK_emv_. In doing so, the two can obtain each other's public key even though the reader is not connected with the FCA. Thus, the card can use PK_*m*_ to decrypt and verify auth_*m*_ (retrieved from Message 7; cf. Figure 4), so as to authenticate a merchant's reader. And the reader can use PK_emv_ to verify the received auth_*p*_ to authenticate the card. As a result, the reader and the card can achieve mutual authentication without connecting their FCA.
2. Confidentiality: since the card is able to obtain the reader's public key without FCA, it can use the key to encrypt the secret value *S*_*p*_ (the first sensitive data in our protocol) and sends the encrypted message to the reader. The reader decrypts the message with its private key SK_*m*_ and then shares the secret *S*_*p*_ with the card. Next, we adopt the current transport layer security (TLS) protocol [45] and use the shared secret to generate a session key TK. Hereafter, all the reader-phone communications are encrypted with the session key TK. In doing so, we guarantee the confidentiality of our user-merchant communications.
3. Replay attacks: our transaction messages are encrypted by TK and TK is generated with the random numbers *R*_*p*_ and *R*_*m*_ created in this session. An attacker cannot replay a message that has been logged in previous sessions because TK changes in every session.
4. Data privacy: because the reader and the card can authenticate each other, and our protocol prevents replay attacks from evading authentication, and we also guarantee the confidentiality of communications, we can assert that a user's sensitive information, such as credit card number in Cert_emv_^iss^ and transaction data in Data_emv_, will not be exposed to an adversary.
5. Integrity: in our mutual authentication, every message is sent with a message authentication code. After the mutual authentication phase, our protocol follows the EMV standard to protect message integrity. Thus, any modifications to the transmitted messages will be detected.
6. Nonrepudiation: since our proposed transaction protocol complies with the EMV standard except that all the reader-phone communications are encrypted with a session key, our proposed protocol achieves nonrepudiation as EMV does.
7. Man-in-the-Middle (MITM) attacks: because all of our reader-phone communications are encrypted, mutual authentication is required before transactions, replay attacks can be detected and prevented, and message integrity is guaranteed; adversaries cannot masquerade as a reader or a phone to launch MITM attacks.
8. Clone attacks: since current EMV protocols do not authenticate a merchant's reader, a malicious merchant may record transaction information and replay it to perform a transaction in other shops [27]. But our protocol can do mutual authentication and prevents replay attacks. So, attackers cannot collaborate with a shop to prerecord transaction messages and then perform a transaction in other shops.
9. Online-equivalent-security offline transaction: in our protocol, before each offline transaction, a reader needs to check the offline certificate that has been associated with a credit card by its issuing bank. Besides, we can perform offline mutual authentication between a user's phone and a merchant's card reader, which guarantees the confidentiality and message integrity in our protocol and prevents MITM attacks. Therefore, the security strength of our offline transactions can be equivalent to that of EMV's online transactions.

Table 1 shows the security comparison between our protocol (denoted as EPMAR) and the original EMV standards. Since static data authentication (SDA) needs an issuing bank to authenticate a credit card, it cannot authenticate a card without Internet connection [17]. Besides, all of EMV's authentication schemes do not require authentication of card readers [17]. As for confidentiality, EMV standards do not encrypt the communications between a card and a reader. Attackers may eavesdrop on the communications and obtain transaction data such as credit card numbers and the expiry dates. Users' data privacy can be infringed and confidentiality compromised. SDA cannot guarantee nonrepudiation of a customer's offline transaction because it cannot authenticate a credit card and verify whether the card has been revoked without online readers [17, 19]. Also, because EMV standards do not authenticate the reader, attackers can masquerade as a card reader or a card to launch MITM attacks. In clone attacks, current EMV protocols do not authenticate a merchant's reader, so a malicious merchant may record transaction information and replay it to perform a transaction in other shops [27]. As EMV standards cannot verify whether a credit card has been revoked during offline transaction, they have to take higher risk in offline transactions.

To summarize, our proposed protocol achieves offline mutual authentication and uses a bank-issued offline certificate to help a merchant verify whether a credit card has been revoked. So, it can prevent certain security threats in offline transactions.

## 4. Performance Analysis

In this section, we use two NFC-enabled phones, E975 LG Optimus G, to act as a customer's phone and a mobile credit card reader in the analysis of computational loads, communication loads, and storage requirements of our protocol. The specifications of these two phones are 1.5 GHz CPU, 2 GB RAM, and Android OS. We adopt Android API to implement our protocol and EMV's client-end programs.

As Table 2 shows, we use different key lengths and the EMV-defined data lengths [17] to compare the storage requirements, communication loads, and computational loads in our protocol and in EMV's three standards SDA, denoted as ES; DDA, ED; and CDA, EC.

The length of Cert_emv_^iss^ is *L*_*r*_ + 42 bytes, where *L*_*r*_ denotes the length of an RSA key. Our offline certificate is supposed to be smaller than EMV's original certificate because it does not include an issuing bank's public key. Still we take Cert_emv_*off*⁡_^iss^ as *L*_*r*_ + 42 bytes, which is the length of an original EMV certificate, to simulate our worst case.

### 4.1. Storage Requirements

If an issuing bank has issued *n* credit cards to a user's phone and there are *m* valid offline certificates stored in the phone, the phone's static, dynamic, and maximum storage requirements are shown in Table 3, where *L*_*a*_ denotes the length of an AES key.

The “Static Memory Requirements” indicates the storage requirements for a phone to store the credit cards and offline certificates. Since our protocol needs the phone to store FCA's public key PK_fca_ and the offline certificate Cert_emv_*off*⁡_^iss^, it requires (*n* + *m*)*L*_*r*_ + 42*m* bytes more than EMV's DDA and CDA. Compared with EMV's SDA, which demands the least storage for its static memory, our scheme requires 42*m* + 42*n* + (3*n* + *m*)*L*_*r*_ more bytes.

The “Dynamic Memory Requirements” in Table 3 shows the maximum memory required for the phone to complete a credit card transaction. Since the phone needs to buffer the largest message (auth_*p*_ in Message 9) before sending it, we add up the length of auth_*p*_ and all the temporary variables (*R*_*p*_, *R*_*m*_, *S*_*p*_, *S*_mas_, Cert_*m*_^acq^, auth_*m*_, and TK) in the protocol to calculate our maximum dynamic memory usage. The result is 146 + 3*L*_*r*_ + *L*_*a*_. Compared with EMV, ours requires 79 + 2*L*_*r*_ + *L*_*a*_ more bytes. Last of all, “Maximum Storage” in Table 3 sums up the static memory and dynamic memory requirements. Compared with EMV, ours requires (3*n* + *m* + 2)*L*_*r*_ + *L*_*a*_ + 42*m* + 42*n* + 79 more bytes. If we use 3072-bit RSA keys and 256-bit AES keys in our protocol, and store 100 credit cards into a phone, it will take 253 Kbytes. It is only 161 Kbytes more than EMV's SDA. The storage requirement is affordable for current smartphones to take 161 Kbytes to run the client end programs of our transaction protocol [46].

In Table 4, we compare our protocol with the EMV standards in terms of static and dynamic memory usage on a merchant's credit card reader, where *f* denotes the number of FCAs' public keys that a reader has.

Table 4 shows that our scheme needs (78 + 4*L*_*r*_)*f* bytes for static memory. This is because the reader that supports our protocol has to store SK_*m*_, Cert_acq_^fca^, and Cert_*m*_^acq^. The reader's maximum dynamic memory usage occurs when it receives Message 9's auth_*p*_. It requires 146 + 3*L*_*r*_ + *L*_*a*_ bytes for its dynamic memory, which is *L*_*r*_ + 42 + *L*_*a*_ bytes more than EMV's SDA. Its maximum storage requirements just increase by 3*fL*_*r*_ + *L*_*a*_ + 78*f* + 43 bytes. If we use 3072-bit RSA keys and 256-bit AES keys in our cryptology and a merchant supports five FCAs (including the most used FCAs), the storage requirement for a card reader to support our protocol is 9.4 Kbytes. It is acceptable for current credit card readers [47].

### 4.2. Communication Loads

In this section, we list the commands used in our protocol and in SDA, DDA, and CDA in a chronological order of their appearance. As Table 5 shows, the main difference between our protocol and EMV is that our protocol requires three more commands for mutual authentication, that is, numbers 4–6, which means six more messages in total (including the response message).

The following analysis demonstrates the extra message length required for our protocol. For mutual authentication, we insert the command READ RECORD as commands number 4 and number 6; see Table 5. Apart from these two commands, our protocol also uses the following commands' RFUs. In command number 1 SELECT, we add two parameters PDOL and *R*_*p*_. In command number 2 GET PROCESSING OPTIONS, we add three parameters *R*_*m*_, Cert_*m*_^acq^, and Cert_acq_^fca^. In command number 5 VERIFY, we use the command to send auth_*m*_ instead of the access code. In command number 7 READ RECORD, we add an offline certificate Cert_emv_*off*⁡_^iss^. We use command number 9 EXTERNAL AUTHENTICATE to send the offline certificate to a user's phone, which increases by *L*_*r*_ + 42 bytes. To sum up, our protocol requires 7*L*_*r*_ + 200 more bytes than the EMV standards.

**Figure 10:**
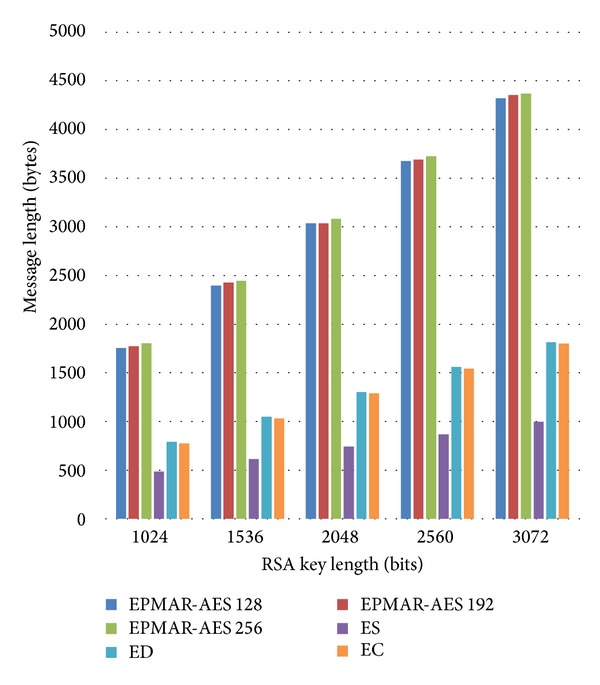
Comparison of message lengths—offline transaction.

**Figure 11:**
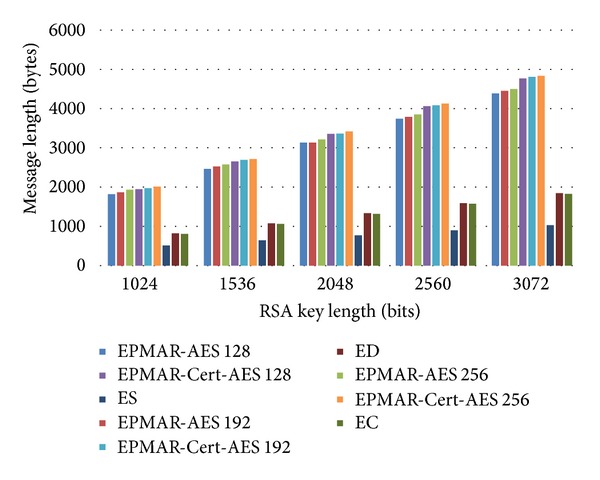
Comparison of message lengths—online transaction.

Because our protocol uses a session key TK to encrypt messages, we analyze the impact of TK's length on protocol performance. With TK of different lengths 128, 192, and 256 bits, we compare the total message length of our scheme with that of other methods in offline transaction (see Figure 10) and in online transaction (see Figure 11). In Figure 10, because our scheme uses AES for encryption, our total message length increases by 30 bytes due to message padding. Besides, the message lengths of our scheme and of EMV standards are proportional to RSA's key lengths. We can see the message lengths increase linearly with RSA's key lengths; see Figure 10.

Figure 11 depicts the total message length of our scheme and of EMV in an online transaction, in which the request for an offline certificate is denoted as EPMAR-Cert. Since the online transaction of both EMV and our scheme is the same as the offline transaction except that they require four extra messages for an offline transaction, the total message length of an online transaction increases by 30 bytes compared to that of an offline transaction with all kinds of RSA key lengths.

Next, we compare the transmission time of our protocol with that of EMV. In our simulation, we activate an NFC phone's card enumeration mode, which acts as a credit card, and we use another NFC phone's read-write mode to act as a credit card reader. According to Mifare DESFire's ISO 14443 standard [48], the tag-reader transmission rate is 858 Kbits/s. We use the transmission rate and the message lengths that we have calculated in Figures 10 and 11 to analyze the transmission time between a credit card and its reader.

As Tables 6 and 7 show, it takes about 200 ms for our scheme to perform one transaction, which is about three times longer than the original EMV standards. In order to prove that our scheme can perform a transaction within the time that EMV suggests, we will analyze our protocol's total time consumption and our computational time in the following subsection.

### 4.3. Computational Loads

In this subsection, we compare the computational loads of our protocol with those of the EMV standards. Because a comparison operator requires only a little computation, compared with a cryptographic algorithm, it will be left out of our performance analysis.

**Figure 12:**
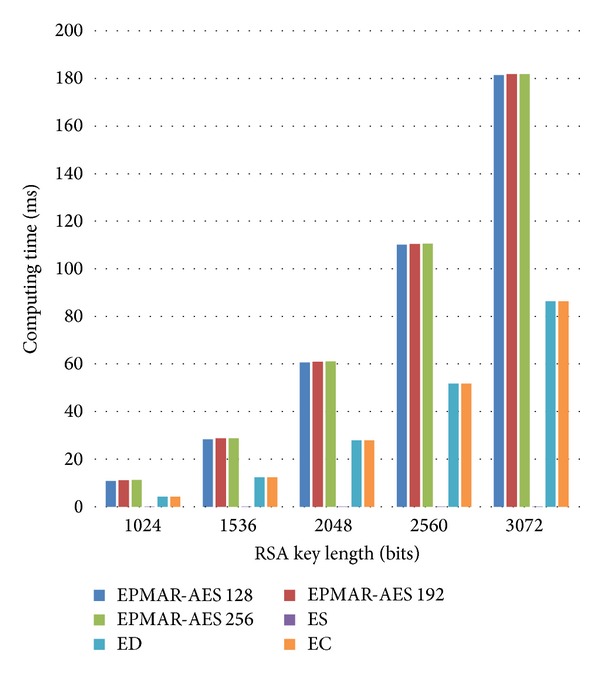
Phone's computational loads—offline transaction.

As Figure 12 shows, the increase of RSA's key length, rather than the increase of AES's key length, has more impact on the computational time for an offline transaction protocol. Because our scheme performs mutual authentication, the computational time of our protocol is about 2.5 times more than CDA's, which requires only one RSA private key encryption. Besides, SDA has the least computation time because it only uses AES to encrypt messages.

**Figure 13:**
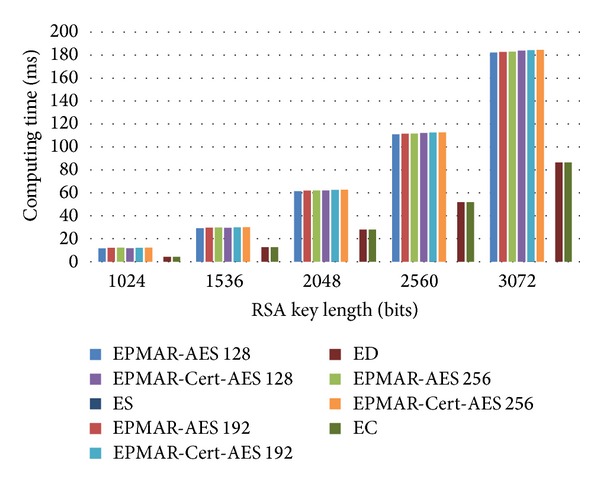
User phone's computational loads—online transaction.

Figure 13 shows the computational loads of a user's phone in an online transaction. During our request of an offline certificate, a user's phone has to take its user-issuer shared key to decrypt and then to verify the received offline certificate. But our offline certificate deployment protocol does not need the phone to execute the second command GENERATE AC. That is to say, the computation time of our certificate deployment protocol is less than that of our online transaction protocol. Since the transaction flow of our online transaction protocol is in accordance with EMV, except all the user-merchant AES encryption, the computation time of our protocol is 2 times more than that of EMV because our protocol needs to use ASE to encrypt every message.

**Figure 14:**
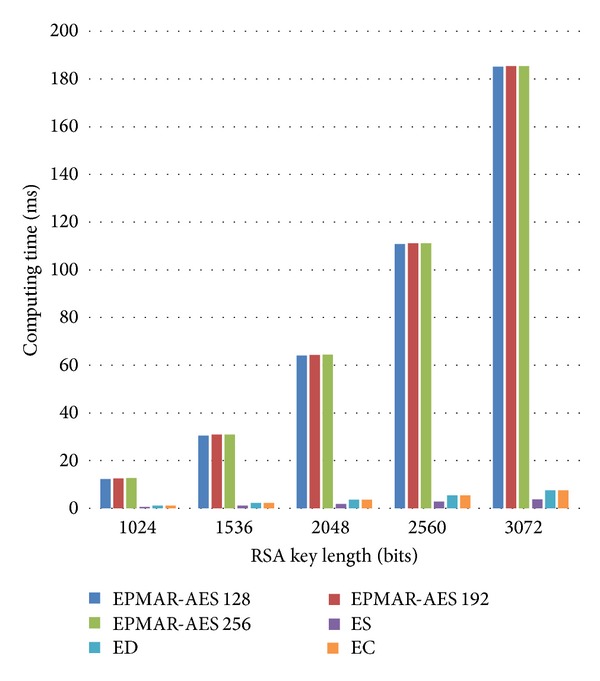
Reader's computational time—offline transaction.

In Figure 14, we find that our card reader has much higher computational loads than EMV's reader in an offline transaction. The main reason is that our mutual authentication needs to run two more RSA private-key cryptographic algorithms and two more RSA public-key cryptographic algorithms.

**Figure 15:**
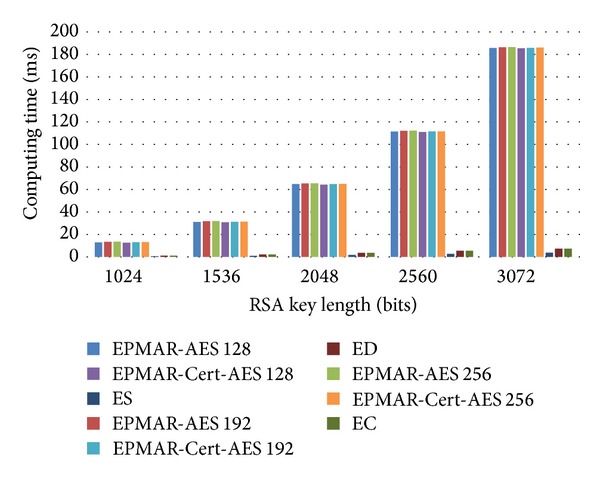
Reader's computational time—online transaction.

Figure 15 shows the comparison of different schemes' computational loads in an online transaction. We find that the computation time of an online transaction is almost the same as that of an offline transaction because those online transaction protocols require only one more message for bank authorization. For them, there is no other encryption/decryption required for further messages.

Since the EMV standards require a transaction to be completed in 500 ms [49], we calculate our phone's and reader's total time consumption in computation and in transmission to prove that our protocol can satisfy EMV's time requirement. The results are shown in Tables 8 and 9. According to the tables, if we use RSA 3072 bits for encryption, it will take our protocol more than 500 ms to perform one transaction. But if we use RSA keys under 2560 bits, our scheme can perform a transaction within 500 ms.

Although our protocol has higher requirements for computation, communication, and storage than the original EMV standards, the increased system loads are still acceptable for current transaction systems. Besides, our simulation uses JAVA's libraries to implement the cryptographic algorithms. If we can run the algorithms with specialized cryptographic hardware, we can decrease the computation time or even use longer keys to secure the communications and transactions.

## 5. Conclusion

Our proposed protocol is able to perform online/offline transaction in compliance with the EMV standards, and our modifications to EMV are transparent to merchants and users. Besides, we prove that our protocol prevents impersonation attacks and avoids eavesdropping on sensitive data because we perform mutual authentication between the phone and the reader and because their communications are encrypted. Also, our scheme resists the security threats in the EMV standards, such as MITM attacks and clone attacks. Although mutual authentication and the added cryptographic algorithms slightly increase the computation, communication, and storage loads, our experiment results show that if we use current EMV's RSA 1024 bits for encryption, our total increased time is about 100 ms. It satisfies EMV's time requirement for a transaction, 500 ms. The increased communication loads in our protocol are also affordable for current smartphones and merchants' reading devices. Moreover, our protocol provides a mechanism to deploy an offline certificate on an NFC phone without interrupting the user at all. The user's issuing bank can evaluate the transaction risk and write rules into the certificate, such as the maximum transaction amount and the expiry time. This certificate serves as the bank's endorsement of an offline transaction. It provides not only online-equivalent risk control for the merchants, but also online-equivalent credits for the users. While customers have more shopping choices, merchants and banks can enjoy more benefits from the transactions.

## Figures and Tables

**Table 1:** Security comparison with current EMV protocols.

	EPMAR	EMV (with CDA)	EMV (with DDA)	EMV (with SDA)
Offline mutual authentication	○	△^∗1^	△^∗1^	×
Confidentiality	○	×	×	×
Replay attacks	○	○	○	○
Data privacy	○	×	×	×
Integrity	○	○	○	○
Nonrepudiation	○	○	○	△^∗2^
MITM attacks	○	×	×	×
Clone attacks	○	×	×	×
Online-level security	○	△^∗3^	△^∗3^	△^∗3^

^∗1^CDA and DDA only authenticate the credit cards; they do not authenticate the readers.

^∗2^SDA can achieve a reader's nonrepudiation, but it does not have a user's nonrepudiation.

^∗3^EMV standards cannot verify whether a credit card has been revoked without readers being online.

**Table 2:** Data lengths (bytes).

Variable name	Length
Data_emv_	*L*_*r*_ + 67
ATC	2
Cert_emv_^iss^, Cert_*m*_^acq^	*L*_*r*_ + 42
Cert_iss_^fca^, Cert_acq_^fca^	*L*_*r*_ + 36
Type	20
*R*_*p*_, *R*_*m*_	4
Req, TC, ARQC, AAC, OCRC	1
SDAD	*L*_*r*_
ACK	1
AIP + AFL	38
ARC	2
Data_cdol1_	45
Data_cdol2_	8
AC1, AC2	8
Cert_emv_off_^iss^	*L*_*r*_ + 42
auth_*p*_, auth_*m*_	*L*_*r*_
*S*_*p*_, *S*_mas_	48
PDOL	34

**Table 3:** Storage requirements of phone (bytes).

Schemes	Storage		
	Static memory requirements	Dynamic memory requirements	Maximum storage
EPMAR	179*n* + 5*nL*_*r*_ + *m*(*L*_*r*_ + 42)	146 + 3*L*_*r*_ + *L*_*a*_	146 + 179*n* + 3*L*_*r*_ + 5*nL*_*r*_ + *m*(*L*_*r*_ + 42) + *L*_*a*_
ES	137*n* + 2*nL*_*r*_	67 + *L*_*r*_	67 + 137*n* + 2*nL*_*r*_ + *L*_*r*_
ED	179*n* + 4*nL*_*r*_	67 + *L*_*r*_	67 + 179*n* + 4*nL*_*r*_ + *L*_*r*_
EC	179*n* + 4*nL*_*r*_	67 + *L*_*r*_	67 + 179*n* + 4*nL*_*r*_ + *L*_*r*_

**Table 4:** Storage analysis of card reader (bytes).

Schemes	Storage		
	Static memory	Dynamic memory	Maximum storage
EPMAR	(78 + 4*L*_*r*_)*f*	146 + 3*L*_*r*_ + *L*_*a*_	(78 + 4*L*_*r*_)*f* + 146 + 3*L*_*r*_ + *L*_*a*_
ES	(*L*_*r*_)*f*	103 + 2*L*_*r*_	(*L*_*r*_)*f* + 103 + 3*L*_*r*_
ED	(*L*_*r*_)*f*	145 + 3*L*_*r*_	(*L*_*r*_)*f* + 145 + 4*L*_*r*_
EC	(*L*_*r*_)*f*	145 + 3*L*_*r*_	(*L*_*r*_)*f* + 145 + 4*L*_*r*_

**Table 5:** Comparison of the number of messages with EMV.

No.	Schemes			
	EPMAR	ES	ED	EC
1	SELECT	SELECT	SELECT	SELECT
2	GET PROCESSING OPTIONS	GET PROCESSING OPTIONS	GET PROCESSING OPTIONS	GET PROCESSING OPTIONS
3	GET DATA	GET DATA	GET DATA	GET DATA
4	READ RECORD			
5	VERIFY		INTERNAL AUTHENTICATE	
6	READ RECORD			
7	READ RECORD	READ RECORD	READ RECORD	READ RECORD
8	GENERATE AC	GENERATE AC	GENERATE AC	GENERATE AC
9	EXTERNAL AUTHENTICATE	EXTERNAL AUTHENTICATE	EXTERNAL AUTHENTICATE	EXTERNAL AUTHENTICATE
10	GENERATE AC	GENERATE AC	GENERATE AC	GENERATE AC

**Table 6:** Comparison of transmission time for an offline transaction (ms).

Schemes	Key lengths				
	RSA 1024 bits	RSA 1536 bits	RSA 2048 bits	RSA 2560 bits	RSA 3072 bits
EPMAR-AES 128	73.74	100.53	127.49	154.36	181.49
EPMAR-AES 192	74.41	101.96	127.49	155.04	182.83
EPMAR-AES 256	75.75	102.63	129.46	156.38	183.51
ES	20.37	25.74	31.12	36.49	41.87
ED	33.22	43.97	54.72	65.47	76.22
EC	32.54	43.29	54.04	64.79	75.54

**Table 7:** Comparison of transmission time for an online transaction (ms).

Schemes	Key lengths				
	RSA 1024 bits	RSA 1536 bits	RSA 2048 bits	RSA 2560 bits	RSA 3072 bits
EPMAR-AES 128	76.47	103.34	131.56	157.09	184.22
EPMAR-AES 192	78.48	106.03	131.56	159.11	186.91
EPMAR-AES 256	81.17	108.05	134.92	161.80	188.92
EPMAR-Cert-AES 128	81.80	111.36	140.93	170.49	200.30
EPMAR-Cert-AES 192	82.81	113.04	141.26	171.50	201.98
EPMAR-Cert-AES 256	84.49	114.05	143.61	173.18	202.99
ES	21.63	27.00	32.38	37.75	43.13
ED	34.48	45.23	55.98	66.73	77.48
EC	33.80	44.55	55.30	66.05	76.80

**Table 8:** Total computational time—offline transaction (ms).

Schemes	Key lengths				
	RSA 1024 bits	RSA 1536 bits	RSA 2048 bits	RSA 2560 bits	RSA 3072 bits
EPMAR-AES 128	97.47	160.59	253.97	378.05	551.83
EPMAR-AES 192	98.74	162.62	254.57	379.33	553.77
EPMAR-AES 256	100.20	163.41	256.66	380.79	554.57
ES	21.07	26.98	33.04	39.35	45.73
ED	38.59	58.65	86.21	122.66	170.07

**Table 9:** Total computational time—online transaction (ms).

Schemes	Key lengths				
	RSA 1024 bits	RSA 1536 bits	RSA 2048 bits	RSA 2560 bits	RSA 3072 bits
EPMAR-AES 128	101.29	164.22	258.52	380.79	554.07
EPMAR-AES 192	104.5	168.11	259.72	384.01	557.96
EPMAR-AES 256	107.43	170.37	263.32	386.94	560.21
EPMAR-Cert-AES 128	106.45	172.34	268.33	395.1	571.56
EPMAR-Cert-AES 192	108.36	174.92	269.56	397.01	574.14
EPMAR-Cert-AES 256	110.22	176.11	272.09	398.87	575.33
ES	22.43	28.34	34.4	40.71	47.09
ED	39.95	60.01	87.57	124.02	171.43
EC	39.27	59.33	86.89	123.34	170.75

## Notations

Cert_target_^publisher^: A certificate issued by a publisher to a target; for example, Cert_iss_^fca^ is issued by a financial certificate authority (FCA) to an issuing bank
PK_target_: A target's public key; for example: PK_iss_ is an issuing bank's public key
SK_target_: A target's private key; for example, SK_iss_ is issuing bank's private key
Kenc_emv_, Kmac_emv_: The symmetric keys shared between an issuing bank and its virtual credit card
*R*_*m*_, *R*_*p*_: Random numbers
*S*_*p*_: A secret value generated by the phone
*S*_mas_: A secret value used to generate the session key TK
TK: A session key used to encrypt messages between a phone and a card reader
*E*_*k*_(*M*): Encryption of message *M* with a key *k*; for example, *E*_Kenc_emv__(*M*) is encryption of message *M* with Kenc_emv_
*D*_*k*_(*M*): Decryption of message *M* with key *k*
MAC_Kmac_emv__(*M*): A function using message *M* and key Kenc_emv_ to calculate message *M*'s message authentication code (MAC)
HMAC_*k*_(*M*): A function using *k* to hash message *M* into a MAC
*H*(*M*): Hash of message *M* as a MAC.
